# Early identification of sepsis-induced coagulopathy in critical ill patients: an analysis from MIMIC IV database

**DOI:** 10.1186/s12879-025-11482-5

**Published:** 2025-08-26

**Authors:** Yiming Dong, Nana Cao, Jinlei Wu, Xilong Liu, Xuyang Ji, Xiaofei Yin, Shuo Wu, Bailu Wang, Shujian Wei, Yuguo Chen

**Affiliations:** 1 Department of Emergency and Chest Pain Center, Qilu Hospital of Shandong University, Jinan, China; 2 Clinical Research Center for Emergency and Critical Care Medicine of Shandong Province, Qilu Hospital of Shandong University, Jinan, China; 3 Key Laboratory of Emergency and Critical Care Medicine of Shandong Province, Qilu Hospital of Shandong University, Jinan, China; 4 Key Laboratory of Cardiopulmonary-Cerebral Resuscitation Research of Shandong Province, Qilu Hospital of Shandong University, Jinan, China; 5 NMPA Key Laboratory for Clinical Research and Evaluation of Innovative Drug, Clinical Trial Center, Qilu Hospital of Shandong University, Jinan, 250012 Shandong China

**Keywords:** Sepsis, Sepsis-induced coagulopathy (SIC), Early identification, MIMIC database

## Abstract

**Background:**

To develop a model for early identification of coagulopathy in septic patients.

**Methods:**

Patients with sepsis were identified from the Medical Information Mart for Intensive Care (MIMIC)-IV database. Patients who did not meet the sepsis-induced coagulopathy (SIC) scoring criteria upon admission but developed SIC within the subsequent 7 days were considered to be in a pre-SIC state at baseline. Baseline clinical features of the patients were screened by lasso regression. Subsequently, these features underwent multivariate logistic regression for model construction, followed by testing the stability of the model in the test set.

**Results:**

A total of 7,806 patients were included in the study from the MIMIC-IV database, comprising 7,080 without SIC and 726 with pre-SIC. Patients with pre-SIC had higher criticality scores compared to patients with Non-SIC. Pre-SIC was identified as an independent risk factor for hospitalization, 28-day, 90-day, and 1-year mortality in patients with sepsis. Patients with pre-SIC who received early heparin had lower 28-day mortality compared to those without treatment. The SIC scoring system demonstrated a sensitivity of 77.0% for identifying pre-SIC, a specificity of 53.9%, and an AUC of 0.694 (95% CI: 0.659–0.730). Based on SIC scoring system, additional clinical features were added to the pre-SIC model, ultimately yielding 70% sensitivity and 76.2% specificity with an AUC of 0.802 (95% CI: 0.773–0.830) in the validation set.

**Conclusion:**

The development of SIC is associated with increased mortality rate in patients with sepsis, and precise identification of this group of patients and individualized treatment may be important for improving prognosis.

**Supplementary Information:**

The online version contains supplementary material available at 10.1186/s12879-025-11482-5.

## Background

Disseminated intravascular coagulation (DIC) is a serious and potentially fatal complication that can occur during the course of sepsis. In clinical practice, the incidence of DIC ranges from 10 to 15% in patients with trauma or cancer and up to 40% in patients with sepsis [1]. Two commonly utilized scoring systems for identifying coagulation abnormalities in septic patients are the sepsis-induced coagulopathy [2] (SIC) and the International Society on Thrombosis and Haemostasis (ISTH) overt DIC scoring systems [3], both recommended by the Scientific and Standardization Committee (SSC). Research indicates that SIC often precedes the onset of overt DIC, suggesting SIC as an early phase of DIC [4]. Moreover, the SIC scoring system exhibits high sensitivity in the early detection of septic coagulopathy [5]. Nevertheless, patients diagnosed with SIC frequently face a notably high mortality rate, and the efficacy of anticoagulation therapy in this population remains inconclusive [6–8]. Therefore, relying solely on the presence of overt coagulopathy may delay timely recognition of high-risk patients. Early identification of subclinical coagulopathy may provide a critical window for intervention and improve clinical outcomes.

Currently, there is limited research on the early identification of patients in the pre-SIC state. Early recognition and personalized treatment are crucial for improving the prognosis of these individuals [3]. Our hypothesis posits that a model utilizing the clinical characteristics of septic patients could effectively identify individuals in the pre-SIC state.

## Methods

### Source of the data

This study conducted a retrospective analysis utilizing the Medical Information Mart for Intensive Care (MIMIC)-IV dataset [9]. MIMIC-IV, an advanced version of MIMIC-III, contains comprehensive, high-quality data on patients admitted to the intensive care units (ICUs) at the Beth Israel Deaconess Medical Center from 2008 to 2019. Access to the MIMIC-IV database was obtained by a single investigator (SW), who was responsible for extracting relevant data (Certification Number: 11,301,845). The methodology and findings of this research adhered to the guidelines outlined in the Transparent Reporting of a multivariate prediction model for Individual Prognosis Or Diagnosis (TRIPOD) statement [10].

### Study population

The inclusion criteria for our study were as follows: age ≥ 18 years, meeting the sepsis 3.0 criteria [11] within 24 h after admission to the ICU, and an ICU stay exceeding 24 h. The exclusion criteria included: presence of SIC on the day of ICU admission; incomplete data on platelet count (PLT), international normalized ratio (INR), and Sequential Organ Failure Assessment (SOFA) score; less than two SIC score assessments within a 7-day period; initiation of continuous anticoagulant therapy before ICU admission; patients with malignant tumors, poisoning, cirrhosis, antiphospholipid syndrome, or other PLT-affecting disorders; pregnancy; and significant outliers in clinical information that could impact data integrity.

### Data collection

Demographic characteristics extracted for analysis included age, gender, and ethnicity. Clinical information encompassed vital signs (respiratory rate (RR), blood pressure, heart rate (HR), oxygen saturation (SpO_2_), temperature), comorbidities (hypertension, diabetes mellitus (DM), ischemic heart disease (IHD), hyperlipidemia, chronic heart failure (CHF), atrial fibrillation (AF), chronic kidney disease (CKD), acute kidney injury (AKI), and chronic obstructive pulmonary disease (COPD) at ICU admission), source of infection, and critical care scores (SOFA, Simplified Acute Physiology Score (SAPS) II, SIC). Baseline interventions included invasive mechanical ventilation (IMV), peripherally inserted central catheter (PICC), renal replacement therapy (RRT), vasoactive agents application, aspirin application, and heparin application. Biochemical parameters analyzed comprised white blood cell (WBC) count, Hemoglobin (Hb), Hematocrit (Hct), PLT, prothrombin time (PT), INR, activated partial thromboplastin time (APTT), serum creatinine (SCR), blood urea nitrogen (BUN), Sodium, Potassium, Calcium, Magnesium, Chloride, potential of Hydrogen (pH), anion gap (AG), bicarbonate, lactate (Lac), and glucose (Glu). Outcomes of enrolled patients included ICU, 28-day, 90-day, and 1-year mortality.

### Diagnosis of pre-SIC

INR, PLT, and adjusted SOFA scores were individually extracted for each day within the first 7 days of ICU admission. The worst result among each day was utilized for SIC score calculation. Sepsis-induced coagulopathy was diagnosed when the total score, combining adjusted SOFA score, PLT, and INR, equaled 4 or more, with the total score of PT and coagulation exceeding 2 [2] (Table S1). Patients who did not meet the SIC scoring criteria upon admission but developed SIC within the consecutive 7 days were considered to be in a pre-SIC state at baseline.

### Statistical analysis

The normal or approximate normal distribution measurement data were expressed as mean ± SD, while measurement data of a non-normal distribution were presented as median (interquartile range). To assess the significance of differences between groups, we utilized Pearson’s chi-square test, independent t-test, and Wilcoxon rank-sum test, as appropriate. Multiple interpolation of missing values was performed using R software. To enhance the robustness of the results, we performed one-to-one nearest neighbor propensity score matching (PSM) with a caliper width of 0.05. Covariates included in the propensity score model—age, race, sex, comorbidities (AF, CKD, IHD, COPD, CHF, AKI), use of IMV, CRRT, vasoactive agents, Lac levels, SIC, SOFA, and SAPS II scores—were selected based on literature consensus. Standardized mean differences (SMDs) were calculated to assess the quality of matching. Kaplan-Meier Curve was calculated to show the mortality of the group of heparin and non-heparin, then log-rank test was performed to assess the significance of differences between groups. Before model construction, the dataset was randomly divided into training and test sets in a 7:3 ratio. In the training set, baseline variables were screened using lasso regression. Variables with *P* < 0.05 and other relevant variables were further analyzed using logistic multivariate analysis. Variables with *P* < 0.05 in the multivariate analysis were then utilized in constructing the pre-SIC model. Specifically, we selected the top four features (PLT, APTT, SAPS II, and vasoactive agents) to construct a simplified pre-SIC identification model. Although the SIC score showed a slightly higher contribution than SAPS II, it was excluded to avoid potential redundancy, since it already incorporates PLT—a variable with a higher independent contribution. Therefore, PLT was retained and the SIC score was excluded to ensure model simplicity and to minimize overlapping effects. The efficacy of the model was validated using the test set. The pre-SIC model was used to generate column-line graphs. Receiver operating characteristic (ROC) curves were employed to calculate the area under the curve (AUC) along with corresponding Hazard Ratio (HR), 95% confidence intervals (CI) and cut-off values. The consistency between observed and predicted results was assessed using calibration curves. Calibration plots were utilized to calibrate the column-line diagram model. Furthermore, the clinical utility of the model and its impact on decision-making were evaluated using decision-curve analysis (DCA). All statistical analyses were performed using R software (v.4.2.0, R Foundation for Statistical Computing). A significance level of *p* < 0.05 was considered statistically significant.

## Results

### Baseline characteristics of patients with sepsis

**Fig. 1:**
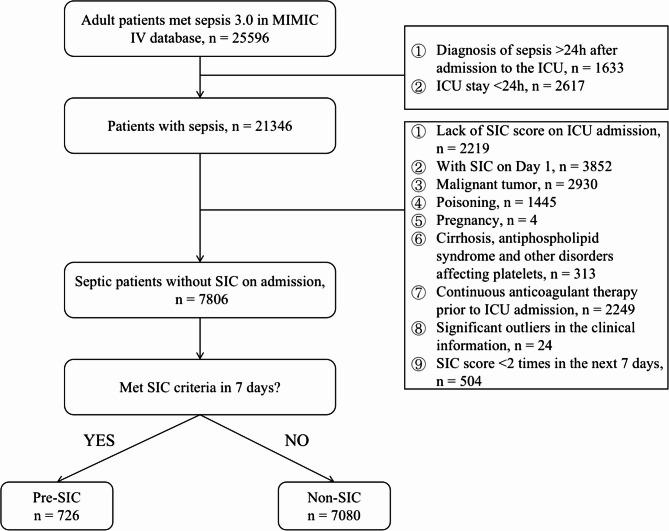
Flow chart of this study

A total of 7,806 patients diagnosed with sepsis were included in the study. Among them, 726 patients progressed to SIC within 7 days of ICU admission, and their admission status was defined as pre-SIC (Fig. 1). Compared with patients who did not develop SIC, patients with pre-SIC were older (66.98 vs. 65.67, *P* < 0.05), had a higher proportion of male patients (64.5% vs. 55.9%, *P* < 0.001). Additionally, they presented with lower body temperature and systolic blood pressure upon admission. Patients with pre-SIC also exhibited higher proportions of comorbidities such as IHD, CHF, CKD, AF, and AKI, while having lower proportions of DM. Furthermore, they had higher admission critical scores including SOFA, SAPS II, and SIC scores. Regarding treatment, a higher percentage of patients with pre-SIC received invasive treatments such as IMV and RRT, as well as non-invasive treatments including vasoactive agents and aspirin. In terms of biochemical parameters, patients with pre-SIC presented with higher levels of SCR, BUN, AG, Lac, potassium, PT, APTT, INR, and Glu, while having lower levels of calcium, WBC count, pH, bicarbonate, and PLT (Table 1).

**Table 1:** Baseline characteristics of septic patients

	Non-SIC	Pre-SIC	*P *value
	*n* = 7080	*n* = 726	
Demographic			
Age, (yrs)	65.67 (16.80)	66.98 (16.07)	0.044
Man, n (%)	3956 (55.9%)	468 (64.5%)	< 0.001
Race, n (%)			0.962
White	4776 (67.5%)	494 (68.0%)	
Black	576 (8.1%)	55 (7.6%)	
Asian	204 (2.9%)	21 (2.9%)	
Other	1524 (21.5%)	156 (21.5%)	
Vital signs on admission			
HR, (bpm)	87.69 (19.21)	89.04 (20.03)	0.072
SBP, (mmHg)	121.30 (24.02)	116.73 (23.99)	< 0.001
DBP, (mmHg)	66.59 (18.06)	65.92 (19.04)	0.351
Body temperature, (°F)	98.23 (1.42)	98.09 (1.59)	0.017
RR, (bpm)	18.00 [14.00, 22.00]	18.00 [14.00, 22.00]	0.096
SpO_2_, (%)	97.31 (4.18)	97.09 (4.60)	0.197
Comorbidity, n (%)			
Hypertension	4787 (67.6%)	491 (67.6%)	1
DM	2325 (32.8%)	206 (28.4%)	0.016
Hyperlipidaemia	2770 (39.1%)	293 (40.4%)	0.543
IHD	1895 (26.8%)	245 (33.7%)	< 0.001
CHF	1337 (18.9%)	188 (25.9%)	< 0.001
CKD	1168 (16.5%)	147 (20.2%)	0.012
COPD	489 (6.9%)	59 (8.1%)	0.251
AF	2089 (29.5%)	330 (45.5%)	< 0.001
AKI	1995 (28.2%)	268 (36.9%)	< 0.001
Source of infection, n (%)			0.738
Pulmonary	2556 (36.1%)	257 (35.4%)	
Other	4524 (63.9%)	469 (64.6%)	
Critical care scores on admission			
SOFA score	3.00 [2.00, 4.00]	3.00 [2.00, 5.00]	< 0.001
SIC score	2.00 [2.00, 3.00]	3.00 [3.00, 4.00]	< 0.001
SAPS II score	35.00 [28.00, 44.00]	39.00 [32.00, 49.00]	< 0.001
Intervention/Treatment on admission, n (%)			
IMV	3460 (48.9%)	478 (65.8%)	< 0.001
PICC	651 (9.2%)	66 (9.1%)	0.98
RRT	370 (5.2%)	84 (11.6%)	< 0.001
Vasoactive agents	2857 (40.4%)	410 (56.5%)	< 0.001
Aspirin	3472 (49.0%)	405 (55.8%)	0.001
Heparin	4885 (69.0%)	492 (67.8%)	0.523
Biochemistry			
WBC count, (×10^3^/µL)	12.20 [9.00, 16.40]	11.80 [8.00, 16.70]	0.034
Hb, (g/dL)	10.82 (2.10)	10.77 (2.34)	0.495
Hct, (%)	32.57 (6.16)	32.52 (7.15)	0.845
PLT, (×10^3^/µL)	207.00 [164.00, 270.00]	165.00 [137.25, 198.75]	< 0.001
PT, (s)	13.80 [12.50, 15.40]	15.40 [13.80, 18.28]	< 0.001
INR	1.20 [1.10, 1.40]	1.40 [1.20, 1.70]	< 0.001
APTT, (s)	29.70 [26.50, 34.60]	33.90 [29.30, 44.08]	< 0.001
SCR, (mg/dL)	1.00 [0.70, 1.40]	1.10 [0.80, 1.70]	< 0.001
BUN, (mg/dL)	18.00 [13.00, 30.00]	21.00 [15.00, 34.00]	< 0.001
Sodium, (mmol/L)	138.79 (5.03)	138.67 (5.79)	0.57
Potassium, (mmol/L)	4.19 (0.71)	4.26 (0.77)	0.006
Calcium, (mmol/L)	8.26 (0.77)	8.14 (1.24)	< 0.001
Magnesium, (mmol/L)	2.01 (0.55)	2.04 (0.56)	0.086
Chloride, (mmol/L)	105.21 (6.58)	105.60 (7.29)	0.128
pH	7.37 (0.09)	7.34 (0.12)	< 0.001
AG, (mmol/L)	14.13 (4.08)	15.08 (4.77)	< 0.001
Bicarbonate, (mmol/L)	23.32 (4.37)	21.76 (4.66)	< 0.001
Lac, (mmol/L)	1.70 [1.20, 2.50]	2.10 [1.40, 3.20]	< 0.001
Glu, (mg/dL)	127.00 [106.00, 162.00]	131.00 [106.00, 175.25]	0.047

*SIC *sepsis-induced coagulopathy, *PSM* propensity score matching, *SMD* standardized mean difference, *HR* heart rate, *SBP* systolic blood pressure, *DBP* diastolic blood pressure, *RR* respiratory rate, *SpO2 *oxygen saturation, *DM* diabetes mellitus, *IHD* ischemic heart disease, *CHF* chronic heart failure, *CKD* chronic kidney disease, *COPD* chronic obstructive pulmonary disease, *AF* atrial fibrillation, *AKI* acute kidney injury, *SOFA* Sequential Organ Failure Assessment, *SAPS* Simplified Acute Physiology Score, *IMV* invasive mechanical ventilation, *PICC* peripherally inserted central catheter, *RRT* renal replacement therapy, *WBC* white blood cell, *Hb* Hemoglobin, *Hct* Hematocrit, *PLT* platelet count, *PT* prothrombin time, *INR* international normalized ratio, *APTT* activated partial thromboplastin time,* SCR* serum creatinine, *BUN* blood urea nitrogen, *pH* potential of Hydrogen, *AG* anion gap, *Lac* lactate, *Glu* glucose

### Survival analysis in septic patients

Patients with pre-SIC demonstrated significantly higher rates of ICU, 28-day, 90-day, and 1-year mortality compared to those without SIC (Table 2). Pre-SIC state at baseline was an independent risk factor for ICU, 28-day, 90-day, and 1-year mortality in patients with sepsis after being adjusted by age, sex, IHD, CKD, baseline SOFA score, SAPS II, IMV, vasoactive agents, and RRT (Table 3).

To further investigate whether patients with pre-SIC state could benefit from early heparin anticoagulation therapy, propensity score matching (PSM) was first performed among pre-SIC patients (Table S2). Subsequently, Kaplan-Meier curves revealed that, among patients with baseline pre-SIC status, those who received heparin therapy had a lower 28-day mortality rate compared to those who did not (*P* < 0.05, Figure S1).

**Table 2:** Outcomes of septic patients

	Non SIC	Pre-SIC	*P* value
	n=7080	n=726	
ICU mortality	653 (9.2%)	163 (22.4%)	<0.001
28-day mortality	848 (12.0%)	181 (24.9%)	<0.001
90-day mortality	1154 (16.3%)	210 (28.9%)	<0.001
1-year mortality	1591 (22.5%)	256 (35.2%)	<0.001

*SIC* sepsis-induced coagulopathy, *ICU* intensive care unit

**Table 3:** Associations between mortality and pre-SIC state of septic patients

Outcomes		Univariable analysis	*P* value	Adjusted multivariable analysis	*P* value
		HR (95% CI)		HR (95% CI)	
ICU mortality		2.310 (1.946-2.743)	<0.001	1.492 (1.249-1.784)	<0.001
28-day mortality		2.271 (1.934-2.666)	<0.001	1.605 (1.360-1.894)	<0.001
90-day mortality		1.975 (1.705-2.287)	<0.001	1.453 (1.249-1.691)	<0.001
1-year mortality		1.775 (1.555-2.025)	<0.001	1.399 (1.222-1.602)	<0.001

*HR* Hazard Ratio, *CI* confidence interval, *ICU* intensive care unit

### Features selection for pre-SIC modeling

**Fig. 2:**
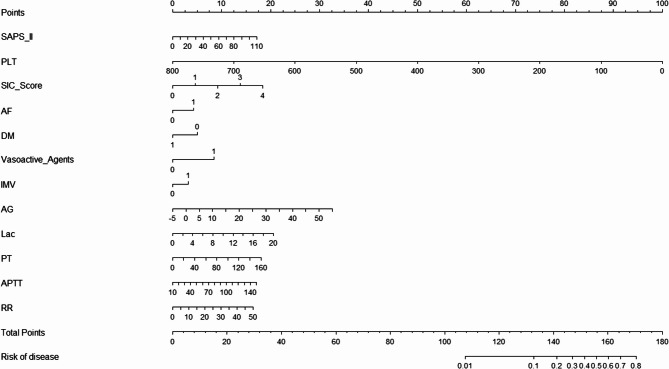
The column-line graph of pre-SIC identification model

SIC, sepsis-induced coagulopathy; RR, respiratory rate; DM, diabetes mellitus; AF, atrial fibrillation; SAPS, Simplified Acute Physiology Score; IMV, invasive mechanical ventilation; PLT, platelet count; PT, prothrombin time; APTT, activated partial thromboplastin time; AG, anion gap; Lac, lactate.

After lasso regression (Table S3, Figure S1), we screened 17 variables, including age, AF, DM, baseline SAPS II score, SIC score, RR, DBP, Glu, PLT, PT, APTT, pH, Lac, AG, bicarbonate, baseline application of IMV and vasoactive agents, for inclusion in logistic multivariate analysis. Ultimately, 12 variables were identified for model construction (Fig. 2, Table S4).

### Comparisons between pre-SIC model and conventional SIC scoring system

**Fig. 3:**
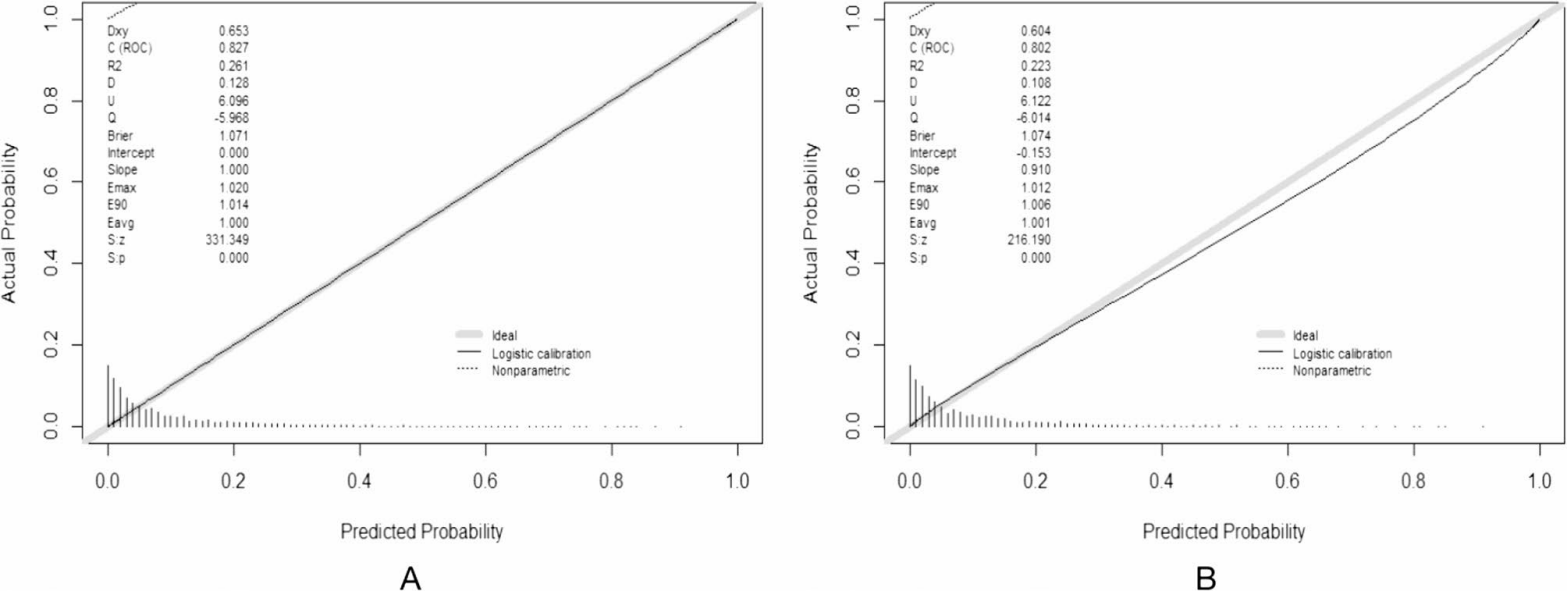
The calibration curves of pre-SIC model in the training set (**A**) and testing set (**B**)

**Fig. 4:**
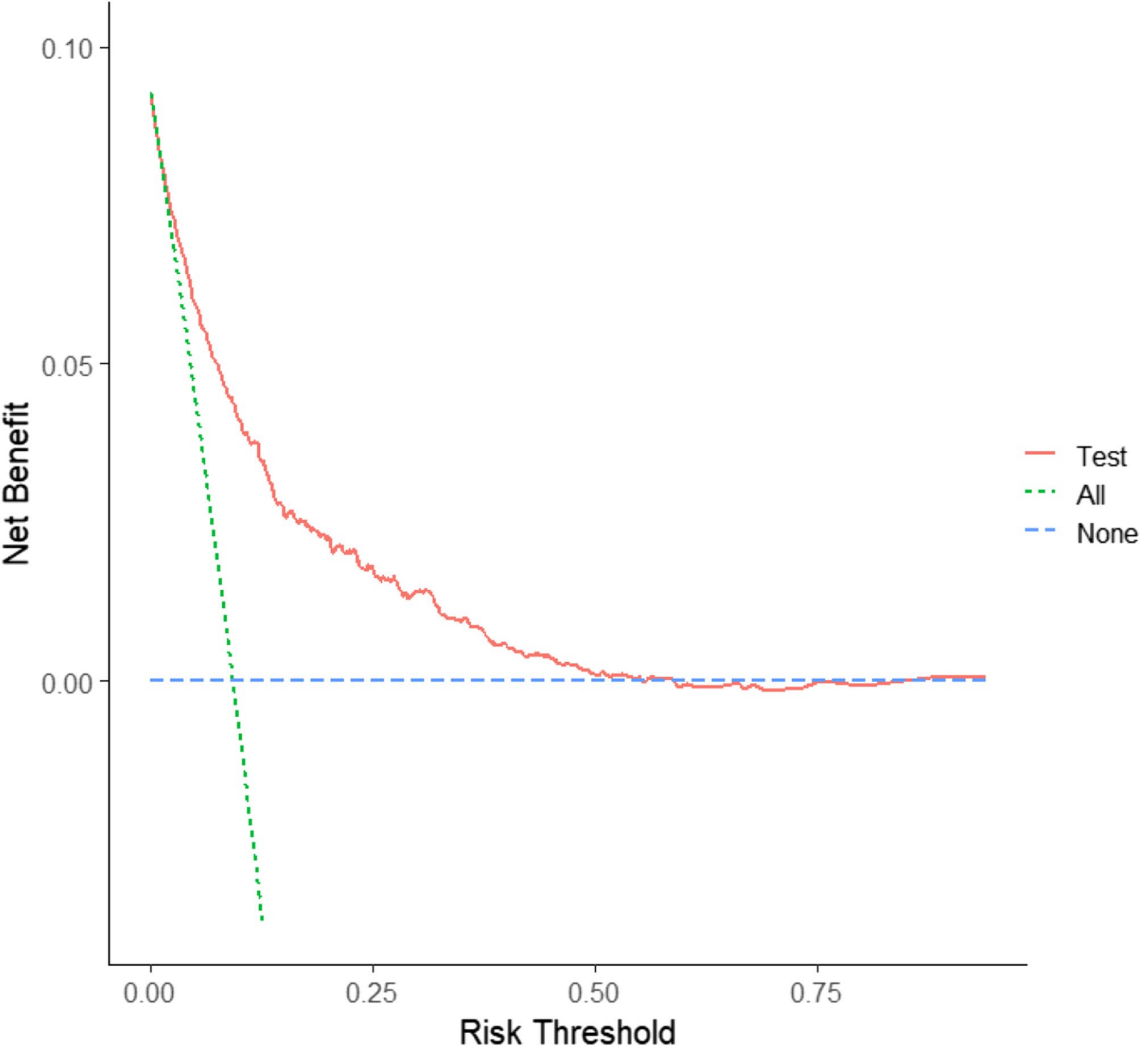
The DCA curve of pre-SIC model in the testing set

In the training set, the pre-SIC classification model obtained a sensitivity of 78.8% and a specificity of 72.0% with an AUC of 0.827 (95% CI: 0.809–0.844). In the test set, the pre-SIC model yielded 70% sensitivity and 76.2% specificity with an AUC of 0.802 (95% CI: 0.773–0.830) (Tables 4 and 5). To assess the performance of the pre-SIC classification model, calibration curves were generated for both the training and test sets, confirming the model’s good fit and stability (Fig. 3). Additionally, DCA based on the column-line graph revealed that at risk thresholds ranging from 0.05 to 0.55, the net return remained greater than 0, indicating clinical utility (Fig. 4)

**Table 4:** Different models of identifying pre-SIC state in the training data

Model	AUC (95% CI)	Sensitivity	Specificity
SIC scoring system	0.691 (0.673-0.720)	78.6%	52.8%
Pre-SIC identification model	0.827 (0.809-0.844)	78.8%	72.0%
Simplified pre-SIC identification model	0.786 (0.767-0.804)	70.5%	73.4%

*HR *Hazard Ratio,* CI *confidence interval, *SIC* sepsis-induced coagulopathy, *AUC* area under the curve

**Table 5:** Different models of identifying pre-SIC state in the test data

Model	AUC (95% CI)	Sensitivity	Specificity
SIC scoring system	0.694 (0.657-0.731)	77.0%	53.9%
Pre-SIC identification model	0.802 (0.773-0.830)	70.0%	76.2%
Simplified pre-SIC identification model	0.752 (0.722-0.783)	73.3%	67.9%

*HR *Hazard Ratio,* CI *confidence interval, *SIC* sepsis-induced coagulopathy, *AUC* area under the curve

**Fig. 5:**
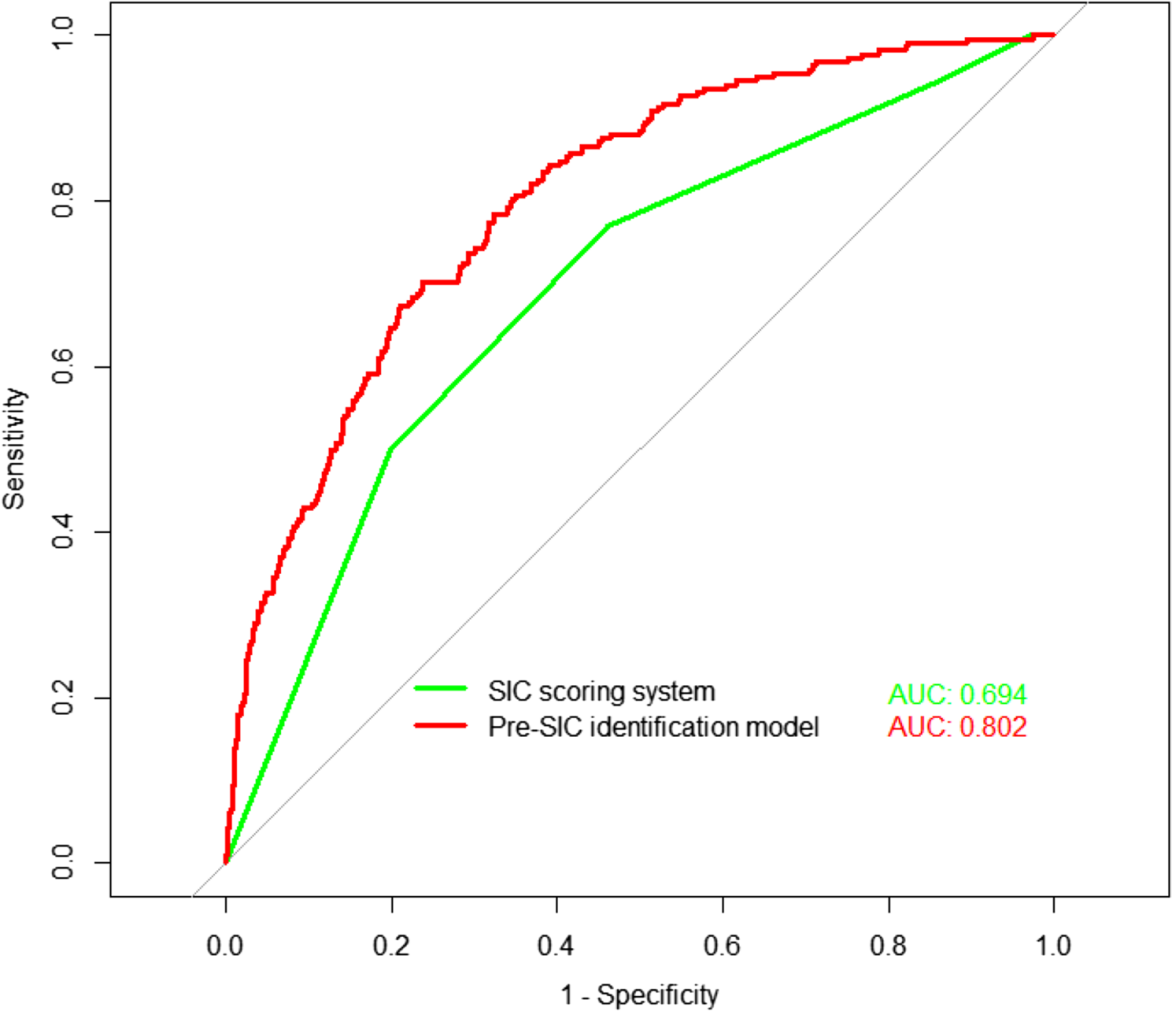
Discriminatory value of SIC scoring system and pre-SIC identification model

We then tested the efficacy of the SIC scoring system for pre-SIC identification and obtained a sensitivity of 77%, a specificity of 53.9% and an AUC of 0.694 (95% CI: 0.657–0.731) in the validation set (Table 5). The efficacy of the pre-SIC classification model based on clinical features was superior to the conventional SIC scoring system (Fig. 5). Moreover, to enhance the generalizability of the model, we further simplified the original model with 12 features by reducing the number of variables and eliminating potential redundancies. Ultimately, based on the coefficients of feature from LASSO regression (Table S3), we selected the top four features (PLT, APTT, SAPS II, and Vasoactive agents) to construct a simplified pre-SIC identification model (Table S4). This simplified model achieved a sensitivity of 73.3%, a specificity of 67.9%, and an AUC of 0.752 (95% CI: 0.722–0.783) in the validation cohort (Tables 4, 5)

### Discussion

Our study found that the development of SIC within 7 days was an independent risk factor for short- and long-term mortality in patients with sepsis. Our study illustrated the efficacy and strong sensitivity of the SIC score in detecting pre-SIC state. Additionally, our model, integrating clinical features with the SIC scoring system, notably enhanced specificity in identifying the targeted population without compromising sensitivity. Some features in the pre-SIC model may be associated with the progression of coagulopathy and do not directly reflect the function of PLT and coagulation. Therefore, relying solely on monitoring indicators such as coagulation function and PLT may have limitations in the early identification of SIC. Furthermore, DIC manifests as highly heterogeneous, with sepsis-induced coagulation dysfunction being distinct from other etiologies of DIC. Notably, SIC seems reversible if timely interventions are applied in the early stages [12]. 

In our study, the ICU mortality rate of patients with pre-SIC was significantly higher than those without SIC. Schmoch et al. [13] reported that the development of coagulopathy was an independent risk factor for 90-day mortality in septic patients based on the SIC scoring system. Therefore, it was not surprising to observe the association of pre-SIC with poor outcomes. Interestingly, there was no significant difference in mortality between patients with baseline SIC and those with pre-SIC (Table S5). Moreover, among patients with pre-SIC state, those who received early heparin therapy had significantly lower 28-day mortality compared to those who did not. These findings suggest that early intervention in pre-SIC may offer clinical benefits and further highlight the clinical importance of early identification of pre-SIC. 

In a large number of studies, anticoagulation has been found to be beneficial in patients with SIC or pre-DIC [14–16], but the mortality rate in this group of coagulopathic patients treated with anticoagulation is still significantly higher compared to those who do not develop significant coagulopathy [13, 17]. This may reflect the inherently more severe systemic pathophysiology in the coagulopathic subgroup, rather than a lack of efficacy of anticoagulation therapy. In addition, lots of studies have explored the efficacy and safety of prophylactic anticoagulation in patients with sepsis, but no uniform answer has been obtained.

The features selected for modeling were derived from routine examinations of septic patients, ensuring accessibility without increasing the economic burden on patients. Notably, several important features associated with the development of coagulopathy in sepsis were identified, including elevated levels of AG and Lac, all of which are closely linked to the pathophysiological process of acidosis. Metabolic acidosis emerges as a common pathophysiological change in patients with sepsis, often concomitant with tissue hypoperfusion, cellular hypoxia, and increased lactate production. The inflammatory response triggered by infection in sepsis can inflict damage on microvascular endothelial cells, leading to vasodilation and increased vascular permeability [18]. These alterations contribute to a reduction in effective circulating blood volume, tissue hypoperfusion, and subsequent cellular hypoxia, culminating in lactic acidosis. The acidic milieu characteristic of acidosis can potentiate thrombin activity, promote thrombin generation, prolong clotting time, and undermine the formation and stability of fibrin [19]. Furthermore, acidosis can foster platelet aggregation and adhesion, further compromising the coagulation capacity of blood [12].

Pre-SIC is indeed a pathophysiological phenomenon of concern in septic patients, and the ability to identify this group of patients at an early stage and provide more precise treatment (such as prophylactic anticoagulation) is important for improving the prognosis of septic patients. In addition, more aggressive volume expansion to correct acidosis and improve electrolyte disturbances in the internal environment may also be helpful in delaying or even reversing the onset of coagulopathy [12, 20].

### Conclusion

In conclusion, the development of SIC is significantly associated with increased mortality in patients with sepsis. Therefore, precise identification of this patient subgroup and implementation of individualized treatment strategies may be crucial for improving prognosis.

### Limitations

We only explored patients within 7 days of ICU admission, there may be a subset of patients who also developed SIC after 7 days but due to this reason we did not record them and categorized them into the Non-SIC group, this subset of patients may have subtle changes in baseline pathophysiological state and may have an impact on the final outcome of our model; compared to the original model, the simplified pre-SIC classification model yielded an AUC of 0.752, which falls below the commonly accepted threshold of 0.8 for good or excellent model performance. Although this version includes significantly fewer features, the reduced predictive accuracy may limit its clinical applicability; in addition, the existence of retrospective studies its inherent limitations, such as the inability to determine causality; due to limitations of the database, we were unable to obtain the levels of fibrinolytic markers such as fibrinogen and D-dimer; furthermore, our results were only internally validated, and an external dataset with a larger sample size is still needed to further validate the stability and generalizability of the model. To address this limitation, we are considering a prospective, multicenter validation study in the future, and we hope to share the findings in subsequent publications.

## Supplementary Information

Supplementary Material 1: Figure S1. Kaplan-Meier Curves of the patients with pre-SIC Figure S2. Lasso regression for features selection Tables S1. SIC scoring system Tables S2. Baseline characteristics of the patients with pre-SIC state Tables S3. Features and coefficients of pre-SIC state after lasso regression Tables S4. Detailed features of the pre-SIC identification model Tables S5. Baseline SIC state and clinical outcomes

## Data Availability

All data generated or analyzed during this study are included in this published article and its supplementary information files.

## Abbreviations

SIC: sepsis-induced coagulopathy
RR: respiratory rate
DM: diabetes mellitus
AF: atrial fibrillation
SAPS: Simplified Acute Physiology Score
IMV: invasive mechanical ventilation
PLT: platelet count
PT: prothrombin time
APTT: activated partial thromboplastin time
AG: anion gap
Lac: lactate
