# Supplementary material for: Early identification of sepsis-induced coagulopathy in critical ill patients: an analysis from MIMIC IV database
Source: BMC Infect Dis. 2025 Aug 26;25:1071. doi: 10.1186/s12879-025-11482-5 (PMC12379342; doi:10.1186/s12879-025-11482-5)
Supplement: Supplementary file 1 — Supplementary Material 1: Figure S1. Kaplan-Meier Curves of the patients with pre-SIC Figure S2. Lasso regression for features selection Tables S1. SIC scoring system Tables S2. Baseline characteristics of the patients with pre-SIC state Tables S3. Features and coefficients of pre-SIC state after lasso regression Tables S4. Detailed features of the pre-SIC identification model Tables S5. Baseline SIC state and clinical outcomes [file 12879_2025_11482_MOESM1_ESM.docx]

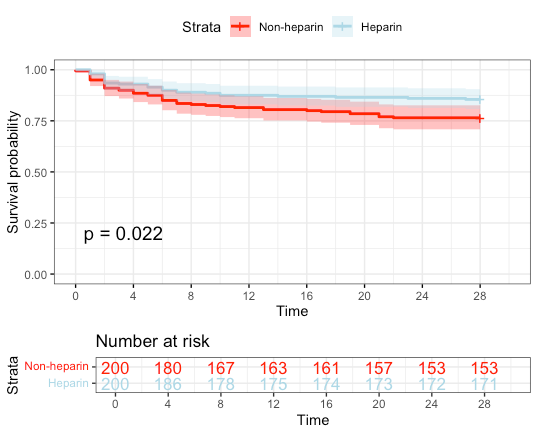


**Figure S1. Kaplan-Meier Curves of the patients with pre-SIC state**


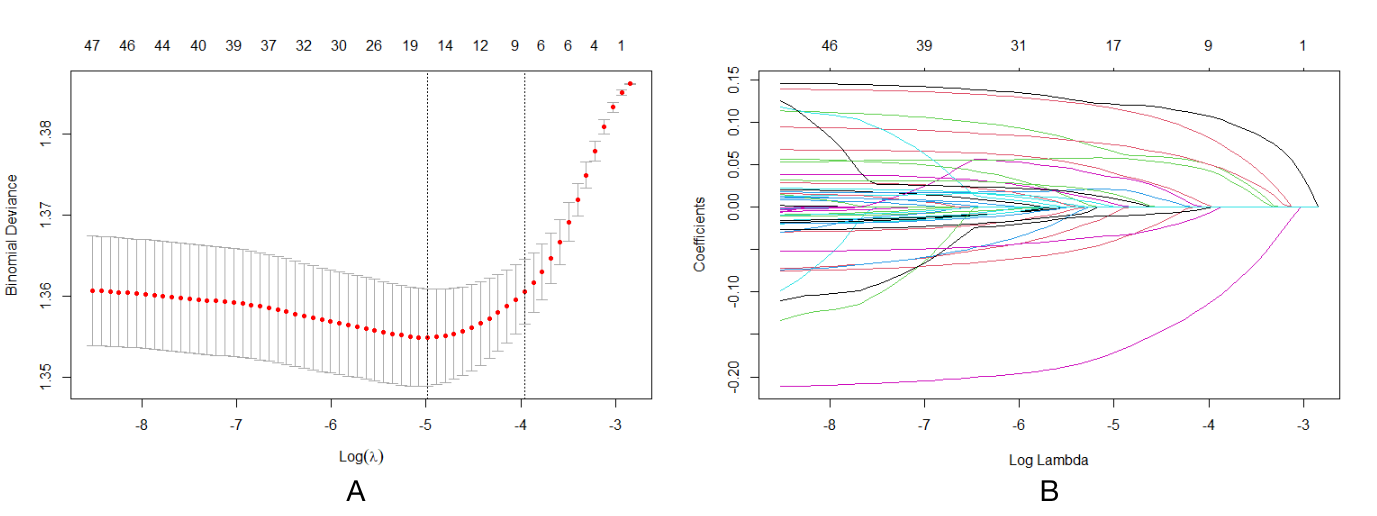


**Figure S2. Lasso regression for features selection**

| **Table S1. SIC scoring system** | | | | |
| --- | --- | --- | --- | --- |
| Category | Parameter | 0 point | 1 point | 2 points |
| Prothrombin time | PT-INR | ≦1.2 | >1.2 | >1.4 |
| Coagulation (×10^9^/L) | PLT | ≧150 | <150 | <100 |
| Total SOFA | SOFA four items | 0 | 1 | ≧2 |

Note: Total SOFA is the sum the four items (respiratory SOFA, cardiovascular SOFA, hepatic SOFA, renal SOFA).

**Abbreviations:** INR, international normalisation ratio; PT, prothrombin time; PLT, platelet count; SOFA, Sequential Organ Failure Assessment.

| **Table S2. Baseline characteristics of the patients with pre-SIC state** | | | | | | |
| --- | --- | --- | --- | --- | --- | --- |
|  | **Before PSM** | | | **After PSM** | | |
|  | Non-heparin | Heparin | SMD | Non-heparin | Heparin | SMD |
|  | n=234 | n=492 |  | n=200 | n=200 |  |
| **Demographic** |  |  |  |  |  |  |
| Age, (yrs) | 64.81 (17.46%) | 68.01 (15.28%) | 0.195 | 64.61 (17.35%) | 66.32 (15.03%) | 0.105 |
| Man, n (%) | 144 (61.5%) | 324 (65.9%) | 0.09 | 125 (62.5%) | 126 (63.0%) | 0.01 |
| Race, n (%) |  |  | 0.148 |  |  | 0.162 |
| White | 163 (69.7%) | 331 (67.3%) |  | 135 (67.5%) | 148 (74.0%) |  |
| Black | 12 ( 5.1%) | 43 ( 8.7%) |  | 11 ( 5.5%) | 11 ( 5.5%) |  |
| Asian | 8 ( 3.4%) | 13 ( 2.6%) |  | 7 ( 3.5%) | 4 ( 2.0%) |  |
| Other | 51 (21.8%) | 105 (21.3%) |  | 47 (23.5%) | 37 (18.5%) |  |
| **Vital signs on admission** |  |  |  |  |  |  |
| HR, (bpm) | 86.93 (17.89) | 90.04 (20.92) | 0.16 | 86.47 (18.45) | 89.72 (20.35) | 0.167 |
| SBP, (mmHg) | 114.47 (22.52) | 118.13 (24.89) | 0.154 | 114.11 (22.63) | 117.75 (23.83) | 0.156 |
| DBP, (mmHg) | 63.66 (18.06) | 67.14 (19.43) | 0.185 | 63.14 (17.30) | 67.26 (19.04) | 0.227 |
| Body temperature, (°F) | 98.09 (1.52) | 98.04 (1.69) | 0.036 | 98.08 (1.56) | 98.03 (1.72) | 0.025 |
| RR, (bpm) | 16.50 [14.00, 22.00] | 18.00 [15.00, 23.00] | 0.185 | 17.00 [14.00, 21.00] | 18.00 [15.00, 21.00] | 0.039 |
| SpO_2_, (%) | 97.91 (3.69) | 96.70 (4.93) | 0.278 | 98.03 (3.10) | 97.25 (3.56) | 0.235 |
| **Comorbidity, n (%)** |  |  |  |  |  |  |
| Hypertension | 144 (61.5%) | 347 (70.5%) | 0.191 | 120 (60.0%) | 132 (66.0%) | 0.125 |
| DM | 53 (22.6%) | 153 (31.1%) | 0.191 | 50 (25.0%) | 60 (30.0%) | 0.112 |
| Hyperlipidaemia | 93 (39.7%) | 200 (40.7%) | 0.018 | 75 (37.5%) | 82 (41.0%) | 0.072 |
| IHD | 56 (23.9%) | 189 (38.4%) | 0.317 | 45 (22.5%) | 50 (25.0%) | 0.059 |
| CHF | 43 (18.4%) | 145 (29.5%) | 0.262 | 37 (18.5%) | 42 (21.0%) | 0.063 |
| CKD | 34 (14.5%) | 113 (23.0%) | 0.217 | 32 (16.0%) | 34 (17.0%) | 0.027 |
| COPD | 13 ( 5.6%) | 46 ( 9.3%) | 0.145 | 12 ( 6.0%) | 13 ( 6.5%) | 0.021 |
| AF | 103 (44.0%) | 227 (46.1%) | 0.043 | 88 (44.0%) | 97 (48.5%) | 0.09 |
| AKI | 53 (22.6%) | 215 (43.7%) | 0.459 | 49 (24.5%) | 48 (24.0%) | 0.012 |
| **Source of infection, n (%)** |  |  | 0.199 |  |  | 0.032 |
| Pulmonary | 68 (29.1%) | 189 (38.4%) |  | 61 (30.5%) | 64 (32.0%) |  |
| Other | 166 (70.9%) | 303 (61.6%) |  | 139 (69.5%) | 136 (68.0%) |  |
| **Critical care scores on admission** |  |  |  |  |  |  |
| SOFA score | 3.00 [2.00, 4.00] | 3.00 [2.00, 5.00] | 0.202 | 3.00 [2.00, 4.00] | 3.00 [2.00, 4.00] | 0.013 |
| SIC score | 3.00 [3.00, 4.00] | 3.00 [3.00, 4.00] | 0.06 | 3.00 [3.00, 4.00] | 3.00 [3.00, 4.00] | 0.023 |
| SAPS II score | 38.00 [31.00, 47.00] | 40.00 [32.75, 51.00] | 0.261 | 38.00 [31.00, 47.25] | 37.00 [30.00, 46.00] | 0.03 |
| **Intervention/Treatment on admission**, n (%) |  |  |  |  |  |  |
| IMV | 147 (62.8%) | 331 (67.3%) | 0.094 | 126 (63.0%) | 122 (61.0%) | 0.041 |
| PICC | 11 ( 4.7%) | 55 (11.2%) | 0.241 | 11 ( 5.5%) | 23 (11.5%) | 0.216 |
| RRT | 9 ( 3.8%) | 75 (15.2%) | 0.395 | 9 ( 4.5%) | 11 ( 5.5%) | 0.046 |
| Vasoactive agents | 164 (70.1%) | 387 (78.7%) | 0.197 | 144 (72.0%) | 140 (70.0%) | 0.044 |
| Aspirin | 127 (54.3%) | 278 (56.5%) | 0.045 | 104 (52.0%) | 104 (52.0%) | <0.001 |
| **Biochemistry** |  |  |  |  |  |  |
| WBC count, (×10^3^/μL) | 11.85 [8.72, 16.20] | 11.80 [7.80, 16.83] | 0.06 | 11.75 [8.50, 16.00] | 11.00 [7.38, 15.07] | 0.214 |
| Hb, (g/dL) | 10.29 (2.33) | 10.99 (2.31) | 0.304 | 10.28 (2.31) | 10.99 (2.20) | 0.318 |
| Hct, (%) | 30.50 (7.11) | 33.48 (6.98) | 0.423 | 30.60 (7.10) | 33.01 (6.47) | 0.354 |
| PLT, (×10^3^/μL) | 154.00 [125.50, 175.75] | 172.00 [146.00, 209.00] | 0.416 | 154.00 [126.50, 178.25] | 166.00 [133.50, 194.25] | 0.203 |
| PT, (s) | 15.60 [14.30, 18.30] | 15.30 [13.57, 18.20] | 0.061 | 15.75 [14.20, 18.42] | 15.10 [13.60, 17.30] | 0.247 |
| INR | 1.40 [1.30, 1.70] | 1.40 [1.20, 1.70] | 0.057 | 1.40 [1.30, 1.70] | 1.40 [1.20, 1.60] | 0.25 |
| APTT, (s) | 32.20 [28.00, 38.08] | 34.80 [29.78, 48.65] | 0.374 | 32.35 [27.90, 38.05] | 33.35 [29.00, 44.02] | 0.214 |
| SCR, (mg/dL) | 0.90 [0.70, 1.28] | 1.20 [0.88, 1.80] | 0.32 | 0.90 [0.70, 1.30] | 1.00 [0.80, 1.40] | 0.031 |
| BUN, (mg/dL) | 17.00 [13.00, 26.00] | 23.50 [16.00, 39.00] | 0.309 | 17.50 [13.00, 27.00] | 20.00 [14.00, 30.00] | 0.023 |
| Sodium, (mmol/L) | 140.00 [137.25, 142.00] | 138.00 [136.00, 141.00] | 0.21 | 140.00 [137.75, 142.00] | 139.00 [136.00, 141.00] | 0.187 |
| Potassium, (mmol/L) | 4.23 (0.66) | 4.28 (0.82) | 0.062 | 4.24 (0.65) | 4.22 (0.78) | 0.017 |
| Calcium, (mmol/L) | 8.15 (1.58) | 8.17 (1.33) | 0.01 | 8.15 (1.69) | 8.14 (1.61) | 0.004 |
| Magnesium, (mmol/L) | 2.14 (0.60) | 2.00 (0.54) | 0.258 | 2.14 (0.61) | 1.97 (0.55) | 0.297 |
| Chloride, (mmol/L) | 107.46 (6.80) | 104.72 (7.36) | 0.387 | 107.06 (7.03) | 105.34 (6.75) | 0.249 |
| pH | 7.36 (0.11) | 7.34 (0.12) | 0.144 | 7.35 (0.12) | 7.35 (0.10) | 0.022 |
| AG, (mmol/L) | 13.77 (4.55) | 15.70 (4.75) | 0.416 | 13.97 (4.75) | 14.53 (4.31) | 0.121 |
| Bicarbonate, (mmol/L) | 22.26 (4.42) | 21.52 (4.76) | 0.159 | 22.26 (4.70) | 22.02 (4.41) | 0.052 |
| Lac, (mmol/L) | 2.25 [1.60, 3.20] | 2.00 [1.30, 3.10] | 0.006 | 2.30 [1.50, 3.12] | 1.90 [1.20, 3.00] | 0.185 |
| Glu, (mg/dL) | 124.00 [104.25, 152.75] | 137.00 [107.00, 182.00] | 0.177 | 124.00 [104.75, 156.00] | 136.50 [107.75, 172.25] | 0.102 |

**Abbreviations:** SIC, sepsis-induced coagulopathy; PSM, propensity score matching; SMD, standardized mean difference; HR, heart rate; SBP, systolic blood pressur; DBP, diastolic blood pressure; RR, respiratory rate; SpO_2_, oxygen saturation; DM, diabetes mellitus; IHD, ischemic heart disease; CHF, chronic heart failure; CKD, chronic kidney disease; COPD, chronic obstructive pulmonary disease; AF, atrial fibrillation; AKI, acute kidney injury; SOFA, Sequential Organ Failure Assessment; SAPS, Simplified Acute Physiology Score; IMV, invasive mechanical ventilation; PICC, peripherally inserted central catheter; RRT, renal replacement therapy; WBC, white blood cell; Hb, Hemoglobin; Hct, Hematocrit; PLT, platelet count; PT, prothrombin time; INR, international normalized ratio; APTT, activated partial thromboplastin time; SCR, serum creatinine; BUN, blood urea nitrogen; pH, potential of Hydrogen; AG, anion gap; Lac, lactate; Glu, glucose.

| **Table S3. Features and coefficients of pre-SIC state afer lasso regression** | |
| --- | --- |
| Features | Coefficients |
| Gender | 0 |
| Age | -0.001492402 |
| SAPS II score | 0.015499189 |
| SOFA score | 0 |
| INR | 0 |
| PLT | -0.041188705 |
| SIC score | 0.029675657 |
| AF | 0.009825728 |
| AKI | 0 |
| CHF | 0 |
| CKD | 0 |
| COPD | 0 |
| IHD | 0 |
| DM | -0.009485589 |
| Hyperlipidaemia | 0 |
| Hypertension | 0 |
| Pneumonia | 0 |
| Aspirin | 0 |
| Heparin | 0 |
| Vasoactive agents | 0.017972404 |
| RRT | 0 |
| IMV | 0.004986137 |
| PICC | 0 |
| HR | 0 |
| SpO_2_ | 0 |
| WBC count | 0 |
| Hct | 0 |
| Hb | 0 |
| Race | 0 |
| AG | 0.008495477 |
| BUN | 0 |
| Calcium | 0 |
| Chloride | 0 |
| SCR | 0 |
| DBP | 8.90E-07 |
| Glu | 0.00070383 |
| Bicarbonate | -0.002626372 |
| Potassium | 0 |
| Lac | 0.013349305 |
| Magnesium | 0 |
| Sodium | 0 |
| pH | -0.008026113 |
| PT | 0.00223637 |
| APTT | 0.027166112 |
| RR | 0.003556383 |
| SBP | 0 |
| Body temperature | 0 |

**Abbreviations:** SIC, sepsis-induced coagulopathy; HR, heart rate; SBP, systolic blood pressur; DBP, diastolic blood pressure; RR, respiratory rate; SpO_2_, oxygen saturation; DM, diabetes mellitus; IHD, ischemic heart disease; CHF, chronic heart failure; CKD, chronic kidney disease; COPD, chronic obstructive pulmonary disease; AF, atrial fibrillation; AKI, acute kidney injury; SOFA, Sequential Organ Failure Assessment; SAPS, Simplified Acute Physiology Score; IMV, invasive mechanical ventilation; PICC, peripherally inserted central catheter; RRT, renal replacement therapy; WBC, white blood cell; Hb, Hemoglobin; Hct, Hematocrit; PLT, platelet count; PT, prothrombin time; INR, international normalized ratio; APTT, activated partial thromboplastin time; SCR, serum creatinine; BUN, blood urea nitrogen; pH, potential of Hydrogen; AG, anion gap; Lac, lactate; Glu, glucose.

| **Table S4. Features of the pre-SIC model** | | | |
| --- | --- | --- | --- |
| Features of the original model | Features of the simplified model | Definitions | Measurement units/scales |
| SAPS II score | SAPS II score | Baseline SAPS II score | / |
| APTT | APTT | Baseline APTT levels | second |
| PT |  | Baseline PT levels | second |
| PLT | PLT | Baseline PLT levels | ×10^3^/μL |
| SIC score |  | Baseline SIC score | / |
| AF |  | AF at baseline | / |
| IMV |  | Receive IMV at baseline | / |
| AG |  | Baseline AG levels | mmol/L |
| Lac |  | Baseline Lac levels | mmol/L |
| RR |  | Baseline respiratory rate | breaths per minute |
| Vasoactive agents | Vasoactive agents | Receive IMV at baseline | / |
| DM |  | DM at baseline | / |

**Abbreviations:** SIC, sepsis-induced coagulopathy; RR, respiratory rate; DM, diabetes mellitus; AF, atrial fibrillation; SAPS, Simplified Acute Physiology Score; IMV, invasive mechanical ventilation; PLT, platelet count; PT, prothrombin time; APTT, activated partial thromboplastin time; AG, anion gap; Lac, lactate.

| **Table S5. Outcomes of septic patients** | | | | |
| --- | --- | --- | --- | --- |
|  | Non SIC | Pre-SIC | SIC | *P* value |
|  | n=7080 | n=726 | n=2123 |  |
| ICU mortality | 653 (9.2%) | 163 (22.4%) | 354 (16.7%) | <0.001 |
| 28-day mortality | 848 (12.0%) | 181 (24.9%) | 511 (24.1%) | <0.001 |
| 90-day mortality | 1154 (16.3%) | 210 (28.9%) | 594 (28.0%) | <0.001 |
| 1-year mortality | 1591 (22.5%) | 256 (35.2%) | 719 (33.9%) | <0.001 |

**Abbreviations:** SIC, sepsis-induced coagulopathy; ICU, intensive care unit.
